# Phenotypic and Genetic Complexity in Pediatric Movement Disorders

**DOI:** 10.3389/fgene.2022.829558

**Published:** 2022-06-01

**Authors:** Min-Jee Kim, Mi-Sun Yum, Go Hun Seo, Tae-Sung Ko, Beom Hee Lee

**Affiliations:** ^1^ Department of Pediatrics, Asan Medical Center Children’s Hospital, Ulsan University College of Medicine, Seoul, South Korea; ^2^ 3billion Inc., Seoul, South Korea; ^3^ Department of Genetics, Asan Medical Center, Ulsan University College of Medicine, Seoul, South Korea

**Keywords:** movement disorders, whole-exome sequencing, next-generation sequencing, neurogenetic disorders, pediatric neurological disease

## Abstract

The complex and evolving nature of clinical phenotypes have made genetically diagnosing pediatric patients with movement disorders difficult. Here, we describe this diverse complexity in the clinical and genetic features of a pediatric cohort examined by whole-exome sequencing (WES) and demonstrate the clinical benefit of WES as a diagnostic tool in a pediatric cohort. We evaluated 75 patients with diverse single or combined movement phenomenologies using WES. WES identified 42 variants in 37 genes (56.0%). The detection rate was highest in patients with dystonia (11/13, 84.6%), followed by ataxia (21/38, 55.3%), myoclonus (3/6, 50.0%), unspecified dyskinesia (1/4, 25.0%), tremor (1/1, 100%), respectively. Most genetically diagnosed patients (90.5%) were affected by other neurologic or systemic manifestations; congenital hypotonia (66.7%), and epilepsy (42.9%) were the most common phenotypes. The genetic diagnosis changed the clinical management for five patients (6.7%), including treatments targeting molecular abnormalities, and other systemic surveillance such as cancer screening. Early application of WES yields a high diagnostic rate in pediatric movement disorders, which can overcome the limitations of the traditional phenotype-driven strategies due to the diverse phenotypic and genetic complexity. Additionally, this early genetic diagnosis expands the patient’s clinical spectrum and provides an opportunity for tailored treatment.

## 1 Introduction

Movement disorders are a heterogeneous group of neurologic disorders characterized by abnormalities in tone, posture, and initiation or control of voluntary movements, as well as unwanted involuntary movement (Sanger et al., 2010; Pearson and Pons, 2019). Pediatric movement disorders frequently present as complex forms in that different movement phenotypes overlap simultaneously or sequentially in the same patients, and typically coincide with other neurodevelopmental symptoms of diverse neurogenetic disorders (Cordeiro et al., 2018; Graziola et al., 2019). Initial manifestations and clinical course can be variable even in common movement disorders with mutations in the same genes. Therefore, reaching an accurate genetic diagnosis and patient-tailored treatment for pediatric disorders sometimes takes a long journey.

Since the advent of massively parallel, genomic-sequencing techniques in the early 2000s, the increased availability, reduced cost, and improved efficiency of next generation sequencing (NGS) have enhanced gene discovery for neurogenetic disorders and facilitated clinicians’ ability make accurate diagnoses (Rexach et al., 2019; Kim et al., 2020). In particular, either whole exome sequencing (WES) or whole genome sequencing (WGS) can reveal more than 5,000 phenotypically and genetically diverse conditions with a single test. Movement disorders are phenomenological, and each one has heterogeneous genetic causes; NGS is definitely more useful for genetic diagnosis than compared with a single gene test (Neveling et al., 2013). Recent genetic diagnosis of pediatric movement disorders by WES has helped clinicians understand their underlying pathophysiology, expand the spectrum of the genotype–phenotype correlation, and provide personalized treatment (Montaut et al., 2018; Graziola et al., 2019; Rexach et al., 2019; Kim et al., 2020; Papandreou et al., 2020). Moreover, movement disorders itself can be used as the key features for genetic diagnosis of pediatric neurological disorders using NGS.

We investigated the diagnostic yield and clinical characteristics of movement disorders to demonstrate the clinical benefit of WES as a diagnostic tool in a pediatric cohort. Furthermore, we tried to delineated these genetic disorders according to their movement phenotype, and systemic or neurological manifestations to attribute the importance of movement phenomenology in the diagnosis of these complex neurogenetic disorders.

## 2 Materials and Methods

### 2.1 Patient Population

We evaluated neurodevelopmental symptoms of a recently built neurodevelopmental cohort for WES of 382 children from the Pediatric Neurology Clinic and Medical Genetics Center at Asan Medical Center Children’s Hospital, Seoul, South Korea, from 2017 to 2020. The neurodevelopmental symptoms in the initial cohort were defined as symptoms that cause neurological referral, such as motor dysfunction, abnormal movement, epilepsy, behavioral problems, macro/microcephaly, or psychomotor delay including failure to achievement of milestone. Pediatric neurologists identified 75 patients with movement disorders as their major symptom in this cohort and those patients were included in this study. We also investigated the clinical features of movement disorders, associated symptoms, previously performed other genetic analyses, neuroimaging tests, and electroencephalographic findings.

### 2.2 Genetic Analysis

We isolated genomic DNA from whole blood or saliva. We then captured the majority of exons in all human genes (approximately 22,000) using a SureSelect Kit (Version C2; Agilent Technologies, Inc, Santa Clara, CA, United States) and sequenced them using a NovaSeq platform (Illumina, San Diego, CA, United States). We then aligned raw genome sequences to a reference sequence (NCBI genome assembly GRCh37; accessed in February 2009). Variants were annotated, filtered and prioritized using software developed in-house, EVIDENCE (3bilion Inc, Seoul, South Korea). EVIDENCE incorporates daily automatically updated databases, variant classification schema based on the ACMG guidelines, and a symptom similarity scoring system as previously described (Kim et al., 2020; Seo et al., 2020; Seo et al., 2022). All identified variants in this study were confirmed by Sanger sequencing and family study. All subjects gave their informed consent for inclusion before they participated in the study. The Institutional Review Board for Human Research at Asan Medical Center approved this study (IRB number 2017-0988).

## 3 Results

### 3.1 Clinical Characteristics of Patients

**FIGURE 1:**
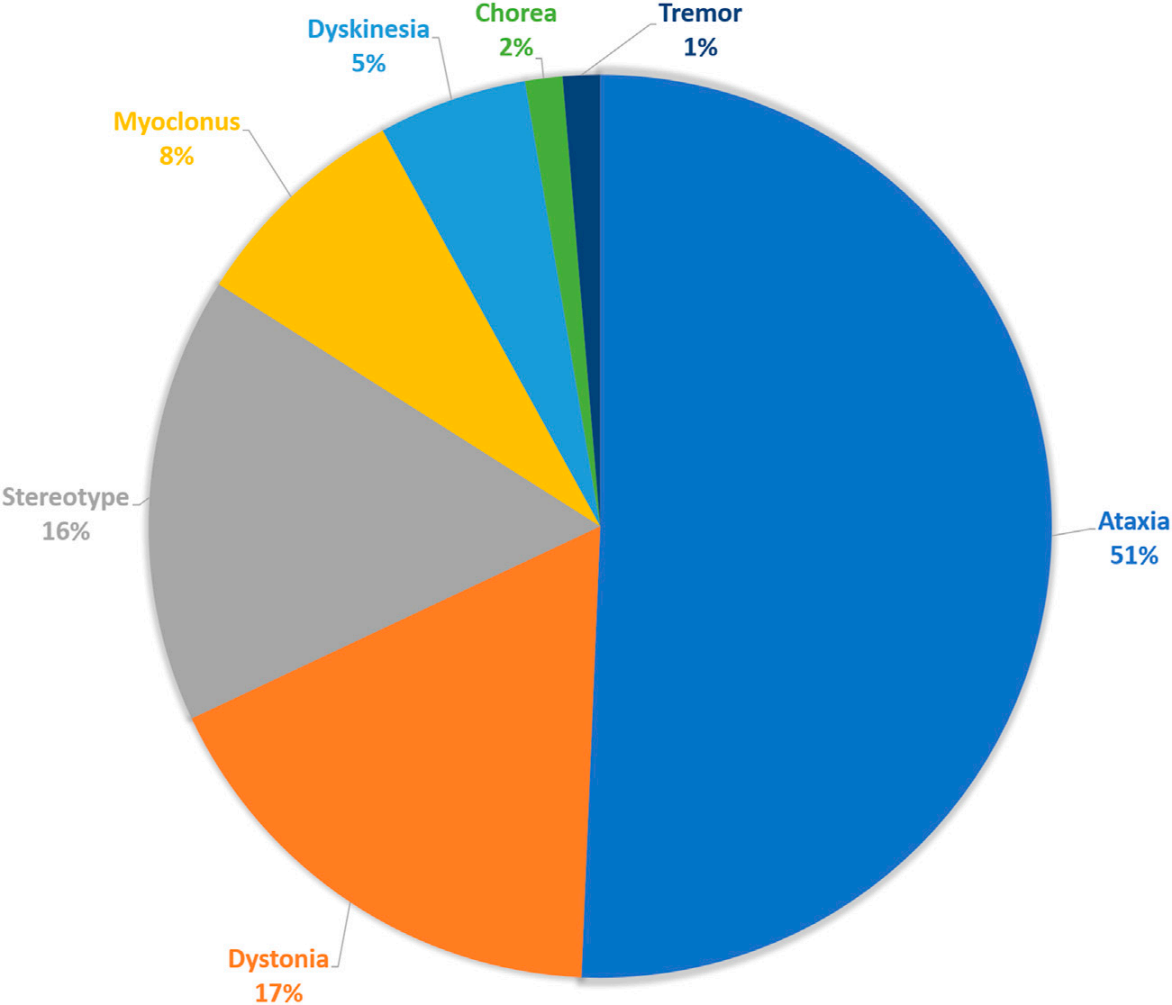
Movement phenomenology. The composition of main movement phenomena of the enrolled patients.

Among the 382 patients of pediatric cohort who performed WES tests for their diagnosis, 75 patients had movement disorders. Prominent movement phenomenology was ataxia in 38 patients (50.6%), dystonia in 13 patients (17.3%), stereotypy in 12 patients (16.0%), myoclonus in 6 patients (8.0%), unspecified dyskinesia in 4 patients (5.3%), or tremor and chorea in 1 patient (1.4%) (Figure 1). Among these, 32 (42.6%) patients had combined movement phenotypes.

Patient demographics, clinical features, and performed tests are described in Table 1. The mean age of symptom onset was 1.8 years, and 66.7% of patients present the movement symptoms before 2 years. Commonly associated neurologic symptoms were congenital hypotonia (42.7%) and epilepsy (34.7%). Of the total patients, 39 (52.0%) showed other systemic symptoms; facial dysmorphism such as prominent or low-set ears, hypertelorism, epicanthal folds, and a flat nasal bridge were most frequently observed in 30.7% of patients, followed by an ocular anomaly (24.0%), and growth delay (14.7%). Before WES, 62 patients (82.7%) underwent genetic testing, including karyotyping, chromosomal microarray (CMA), and single-gene tests, which revealed no specific diagnosis. Additional neuroimaging and EEG study were performed in 90.7% (*n* = 68/75) and 62.7% (*n* = 47/75) of patients, respectively. Partial or complete agenesis of the corpus callosum was the most common abnormality observed in neuroimaging (*n* = 32/43, 74.4%), followed by cerebellar hypoplasia or atrophy (*n* = 10/43, 23.3%), and malformation of cortical dysplasia (4/43, 9.3%). Epileptiform discharges were observed in 72.3% (*n* = 34/47) of patients on the EEG.

**TABLE 1:** Clinical characteristics of patients with movement disorders.

Variables	Total patients N = 75
Gender, n (%)	
Male	40 (53.3)
Female	35 (46.7)
Age at time of whole-exome sequencing	
Mean ± SD, years	5.3 ± 5.2
Onset age of movement symptom	
Mean ± SD, years	1.8 ± 3.0
<2 years old, n (%)	50 (66.7)
Perinatal history, n (%)	18 (25.7)
IUGR	10 (14.3)
Meconium aspiration syndrome	6 (8.6)
Prematurity	4 (5.7)
Oligohydramnios	2 (2.9)
Ventriculomegaly	2 (2.9)
Club foot	1 (1.4)
Cerebellar hypoplasia	1 (1.4)
Associated neurologic symptom, n (%)	47 (62.7)
Congenital hypotonia	32 (42.7)
Epilepsy	26 (34.7)
Microcephaly	15 (20.0)
Macrocephaly	4 (5.3)
Hemiplegia	
Systemic symptom, n (%)	39 (52.0)
Head or neck, including facial dysmorphism	23 (30.7)
Ocular system	18 (24.0)
Growth	11 (14.7)
Hearing loss	4 (5.3)
Musculoskeletal and limb system	3 (4.0)
Cardiovascular system	3 (4.0)
Gastrointestinal system	2 (2.7)
Genitourinary system	2 (2.7)
Hematologic system	2 (2.7)
Endocrinologic system	2 (2.7)
Respiratory system	1 (1.3)
Previous genetic analysis, n (%)	62 (82.7)
Karyotype	56 (74.7)
FISH	4 (5.8)
MLPA	33 (44.0)
CMA	25 (33.3)
Single gene test	21 (28.0)
Targeted exome sequencing or panel test	2 (2.9)
Mitochondrial full genome sequencing	10 (13.3)
Brain MRI, n (%)	68/75 (90.7)
Abnormal findings	43/68 (63.2)
Partial or agenesis of corpus callosum	32/43 (74.4)
Cerebellar hypoplasia or atrophy	10/43 (23.3)
Malformation of cortical dysplasia	7/43 (16.3)
Abnormalities in white matter	6/43 (13.9)
EEG, n (%)	47/75 (62.7)
Epileptiform discharges	34/47 (72.3)

CMA, chromosomal microarray; EEG, electroencephalogram; FISH, fluorescence *in situ* hybridization; IUGR, intrauterine growth restriction; MLPA, multiplex ligation-dependent probe amplification; MRI, magnetic resonance imaging; SD, standard deviation.

### 3.2 Diagnostic Yield and Identified Genetic Variants

**FIGURE 2:**
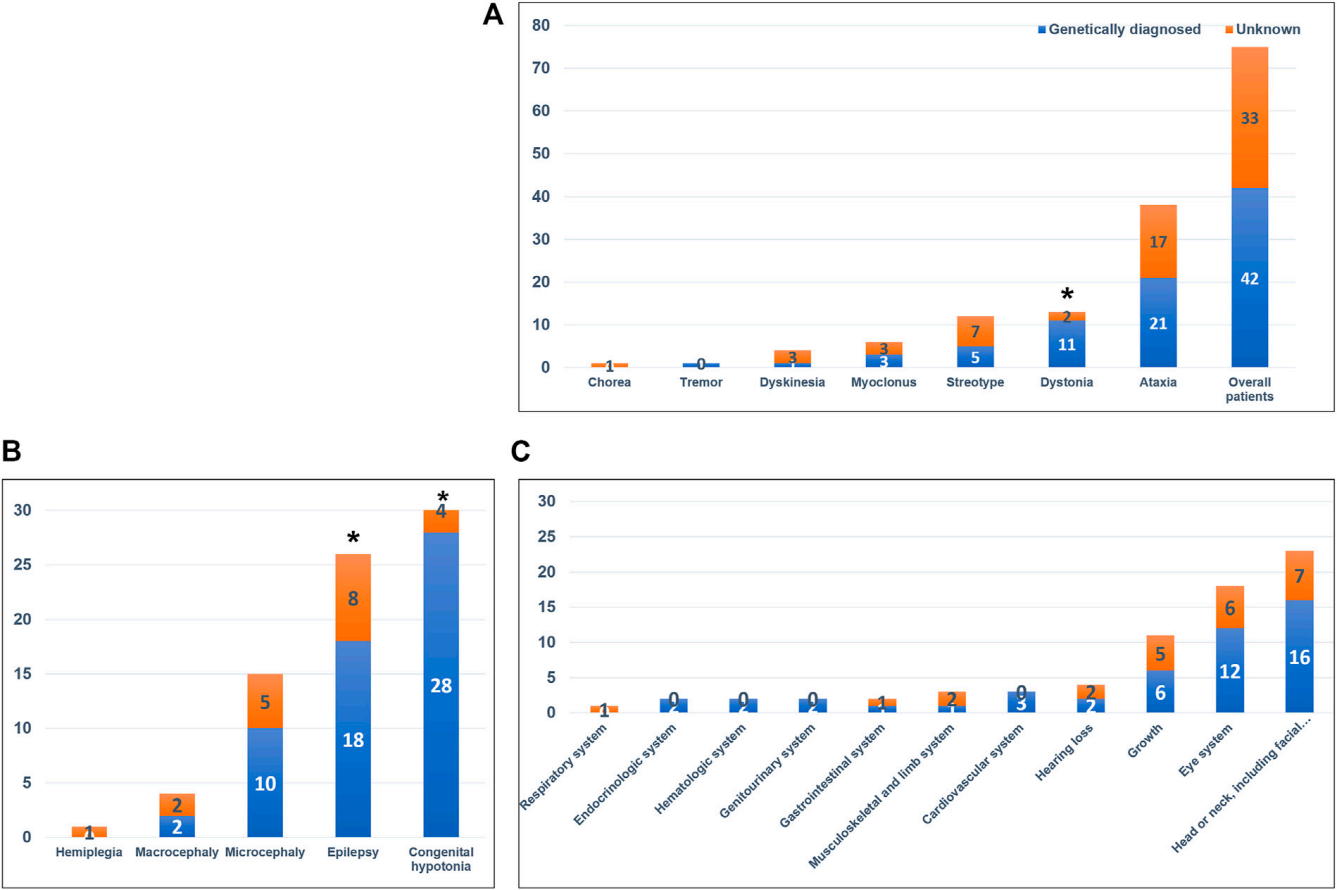
Diagnostic yield of whole-exome sequencing and associated neurologic and systemic symptoms. **(A)** Diagnostic yield in the overall patient cohort and in the different movement disorders. Patients with dystonia had a significantly higher rate of genetic diagnosis compared with patients with other movement disorders (*p = 0.034*). **(B)** Associated neurologic symptoms in the overall patient cohort. Patients with congenital hypotonia (*p < 0.001*) and epilepsy (*p = 0.017*) had a significantly higher rate of genetic diagnosis. **(C)** Associated systemic symptoms in the overall patient cohort. *p-value < 0.05.

We identified 42 variants in 37 genes from the 75 patients and diagnostic yield of patients with movement disorders was 56.0% (42/75; Figure 2 and Table 2). There were no patients with specific family history. Eleven variants (26.2%) were pathogenic, 18 (42.8%) were likely pathogenic, and 13 (31.0%) were variants of uncertain significance (VUS). Patients with VUS were diagnosed with a neurodevelopmental disease according to the results of their parent’s tests, inheritance, and matched clinical phenotype. There were no statistical differences in diagnostic yield between patients who had performed previous genetic tests and those who did not (*n* = 7/13, 53.8% vs *n* = 37/62, 59.7%, *p = 0.464*). Variants were detected in 21 of 38 patients with ataxia (55.3%), 11 of 13 patients with dystonia (84.6%), 5 of 12 patients with stereotypy (41.6%), 3 of 6 patients with myoclonus (50.0%), 1 of 4 patients with unspecified dyskinesia (25.0%), and 1 of 1 patient with tremor (100%), respectively. In regards to prominent movement symptoms, the patients with dystonia had a significantly higher rate of genetic diagnosis compared to patients with other movement disorders (*n* = 11/13, 84.6% vs n = 2/13, 15.4%, *p = 0.034*). Among clinical manifestations other than movement symptoms, epilepsy (18/26, 69.2% vs 8/26, 30.8%, *p = 0.017*), and congenital hypotonia (28/32, 87.5% vs 4/32, 12.5%, *p < 0.001*) was more frequent in patients with a genetic diagnosis. The presence of systemic symptoms was not significantly associated with the genetic diagnosis rate.

**TABLE 2:** Identified variants in our cohort classified on the basis of prominent movement disorders.

	–	Gene (OMIM number)	Inheritance	DNA variation	Amino-acid change	Allele transmission	ACMG guideline	Final diagnosis	Movement phenotype	Concomitant disease
Ataxia	4	GNB1 (NM_002074.)	AD	c.239T>A	p.Ile80Asn	Het De novo	P	Mental retardation, autosomal dominant 42	Persistent ataxia	Congenital hypotonia, short stature, exotropia
	13	KMT2A (NM_001197104.1)	AD	c.3634+1G>A	Splicing	Het De novo	LP	Wiedemann–Steiner syndrome	Persistent ataxia	Congenital hypotonia, dysmorphic face, bilateral staphyloma, ptosis,3rd finger contracture, VUR Gr IV
	24	UBE3A (NM_000462.3)	AD	c.328delG	p.Glu110ArgfsTer2	Het De novo	LP	Angelman syndrome	Persistent ataxia, stereotype, myoclonus	Congenital hypotonia, dysmorphic face, microcephaly, epilepsy
	27	GABRG2 (NM_000816.2)	AD	c.316G>A	p.Ala106Thr	Het De novo	LP	Epileptic encephalopathy, early infantile, 74	Persistent ataxia, tremor	Congenital hypotonia, microcephaly, SNHL, epilepsy, thin CC
	29	SURF1 (NM_003172.3)	AR	c.283del c.367_368del	p.Glu95SerfsTer18 p.Arg123GlyfsTer4	Het From father From mother	LP LP	Leigh Syndrome	Persistent ataxia	T2 HSI in the cerebellum and brainstem
	41	CACNA1A (NM_023035.2)	AD	c.677T>C	p.Leu226Ser	HetDe novo	VUS	Familial hemiplegic migraine 1, Episodic ataxia	Episodic ataxia	Congenital hypotonia, recurrent stroke episode, epilepsy
	73	TCF4 (NM_001243226.2)	AD	c.2044C>T	p.Arg682Trp	Het De novo	P	Pitt–Hopkins syndrome	Persistent ataxia, myoclonus	Congenital hypotonia, dysmorphic face, pulmonary stenosis
	80	ITPR1 (NM_001168272.1)	AD	c.805C>T	p.Arg269Trp	Het De novo	LP	Spinocerebellar ataxia 29	Persistent ataxia, chorea	Congenital hypotonia, macrocephaly, mild cerebellar atrophy
	88	FRRS1L (NM_014334.3)	AR	c.615G>T	p.Met205Ile	Homo From both	VUS	Epileptic encephalopathy, early infantile, 37	Persistent ataxia, dyskinesia, spasticity, chorea	IUGR, congenital hypotonia, epilepsy
	96	FTL (NM_000146.3)	AD	c.485_489dup	p.Glu164TrpfsTer30	Het De novo	LP	Neurodegeneration with brain iron accumulation 3	Progressive ataxia, chorea	Diffuse cerebellar atrophy
	104	SLC2A1 (NM_006516.2)	AD	c.100A>G	p.Asn34Asp	Het De novo	LP	GLUT1 deficiency	Persistent ataxia	Congenital hypotonia
	107	CACNA1A (NM_023035.2)	AD	c.4561G>A	p.Gly1521Arg	Het De novo	VUS	Familial hemiplegic migraine 1, Episodic ataxia,	Episodic ataxia, dystonia	Congenital hypotonia, recurrent stroke episode, epilepsy
	168	MAST1 (NM_014975.2)	AD	c.1549G>A	p.Gly517Ser	Het De novo	VUS	Mega-corpus callosum syndrome with cerebellar hypoplasia and cortical malformations: MCCCHCM	Persistent ataxia	Congenital hypotonia, pontocerebellar hypoplasia
	196	MED13L (NM_015335.4)	AD	c.2239-11T>G	Splicing	Het De novo	VUS	Mental retardation and distinctive facial features with or without cardiac defects	Persistent ataxia	Congenital hypotonia, epilepsy
	252	GNB1 (NM_002074.4)	AD	c.239T>C	p.Ile80Thr	Het De novo	P	Mental retardation, autosomal dominant 42	Persistent ataxia limb spasticity	Congenital hypotonia, dysmorphic face
	288	DYNC1H1 (NM_001376.4)	AD	c.12214G>T	p.Gly4072Cys	Het De novo	VUS	Mental retardation, autosomal dominant 13	Persistent ataxia limb spasticity	Congenital hypotonia, dysmorphic face, polymicrogyria
	275^a^	DEPDC5 (NM_001242896.1)	AD	c.3994C>T	p.Arg1332Ter	Het De novo	c.3994C>T	Epilepsy, familial focal, with variable foci 1	Persistent ataxia	Congenital hypotonia, epilepsy, strabismus, mild cerebellar atrophy
	276	RTEL1 (NM_001283009.1)	AR	c.2141+5G>A c.3994C>T	Splicing p.Arg1332Te	Het From father From mother	c.2141+5G>A c.3994C>T	Hoyeraal-Hreidarsson syndrome	Persistent ataxia, tremor	Congenital hypotonia, microcephaly, dysmorphic face, esophageal web, pancytopenia, small cerebellum and pons
	282	KCNA1 (NM_000217.2)	AD	c.913C>T	p.Leu305Phe	Het From mother	LP	Episodic ataxia/myokymia syndrome	Episodic ataxia	None
	328	ATM (NM_000051.3)	AR	c.8464_8467del c.3024_3025del	p.Asp2822PhefsTer34 p.Asn1010HisfsTer37	Het From father From mother	LP LP	Ataxia telangiectasia	Persistent ataxia	Congenital hypotonia, café au lait spot, cerebellar atrophy
	342^a^	EBF3 (NM_001005463.2)	AD	c.548_551del	p.Ile183ThrfsTer5	Het De novo	LP	Hypotonia, ataxia, and delayed development syndrome	Persistent ataxia	Congenital hypotonia, macrocephaly, café au lait spot, subependymal heterotopia
Dystonia	82	PIGA (NM_002641.3)	XLR	c.986T>C	p.Val329Ala	Hemi De novo	VUS	Multiple congenital anomalies-hypotonia-seizures syndrome 2	Infancy, Generalized, sustained dystonia	EIEE
	93	TCF4 (NM_001243226.2)	AD	c.1296+1G>T	Splicing	Het De novo	LP	Pitt–Hopkins syndrome	Infancy, Generalized, sustained dystonia	Congenital hypotonia, dysmorphic face, microcephaly
	171	CHRNG (NM_005199.4)	AR	c.428C>G c.239_240T	p.Pro143Argsplicing	Het From father From mothe	VUS LP	Escobar syndrome	Infancy, Generalized, sustained dystonia	Dysmorphic face, arthrogryposis
	192	SLC16A2 (NM_006517.4)	XLD/XLR	c.607del	p.Ile203PhefsTer64	Hemi From mother	LP	Allan-Herndon-Dudley syndrome	Infancy, Generalized, sustained dystonia, spasticity, myoclonus	Dysmorphic face, congenital hypotonia, delayed myelination
	206	TOR1A (NM_000113.2)	AD	c.907_909del	p.Glu303del	HetFrom mother	LP	DYT1, early-onset isolated dystonia	Childhood, Focal (right arm and leg) dystonia, aggravated by exercise	None
	219	KCNQ2 (NM_172107.3)	AD	c.901G>A	p.Gly301Ser	Het De novo	P	Epileptic encephalopathy, early infantile, 7	Infancy, Generalized, sustained dystonia	Congenital hypotonia, epilepsy, strabismus, thin CC
	232	NACC1 (NM_052876.3)	AD	c.892C>T	p.Arg298Trp	Het De novo	LP	Neurodevelopmental disorder with epilepsy, cataracts, feeding difficulties, and delayed development	Infancy, Generalized, sustained dystonia, spasticity, chorea	Congenital hypotonia, epilepsy, strabismus, polymicrogyria
	239	SHANK3		22q21.1x3, 22q13.33x1	—	—	—	Phelan-McDermid syndrome	Infancy, Generalized, sustained dystonia, ataxia, chorea	IUGR, Congenital hypotonia, microcephaly, epilepsy, strabismus, leukodystrophy
		ARSA (NM_001085426.2)	AR	c.1238A>G	p.Asp413Gly	Hemi From father	VUS	Metachromatic leukodystrophy		
	263	KMT2B (NM_014727.2)	AD	c.7984C>T	p.Arg2662Trp	Het De novo	VUS	Dystonia 28, childhood-onset	Childhood, Focal (both leg) progressive dystonia	None
	270	WASHC5 (NM_014846.3)	AD	c.1442A>G	p.Lys481Arg	Het De novo	VUS	Spastic paraplegia 8, autosomal dominant	Childhood, Focal (both leg) progressive dystonia,Aggravated by excercise, spasticity	Epilepsy
	329	CTNNB1 (NM_001904.3)	AD	c.1543C>T	p.Arg515Ter	Het De novo	P	Neurodevelopmental disorder with spastic diplegia and visual defects Mental retardation, autosomal dominant 19	Infancy, Generalized, sustained dystonia	Congenital hypotonia, microcephaly Growth retardation, strabismus, TOF
Stereotypy	37	DHDDS (NM_024887.3)	AD	c.632G>A	p.Arg211Gln	Het De novo	P	Developmental delay and seizures with or without movement abnormalities	Infancy, dystonia	SNHL, epilepsy
	209	ARCN1 (NM_001655.4)	AD	c.234_236del	p.Glu78del	Het De novo	VUS	Short stature, rhizomelic, with microcephaly, micrognathia, and developmental delay (SRMMD)	Childhood, dyskinesia, spasticity	IUGR, congenital hypotonia, microcephaly, dysmorphic face, epilepsy, left isomerism, focal cortical dysplasia
	223	ZEB2 (NM_014795.4)	AD	c.73+1G>A	Splicing	Het De novo	LP	Mowat-Wilson syndrome	Childhood	Congenital hypotonia, dysmorphic face, microcephaly, dysgenetic CC
	245	CTNNB1 (NM_001098209.1)	AD	c.163G>T	p.Glu55Ter	Het De novo	LP	Neurodevelopmental disorder with spastic diplegia and visual defects Mental retardation, autosomal dominant 19	Infancy, Spasticity, Dystonia, Chorea	None
	340	MECP2 (NM_004992.3)	XLD	c.502C>T	p.Arg168Ter	Het De novo	P	Rett syndrome	Infancy	Dysmorphic face
Myoclonus	14	TUBB2B (NM_178012)	AD	c.683T>C	p.Leu228Pro	Het De novo	VUS	Cortical dysplasia, complex, with other brain malformations 7	Infancy, Nystagmus	Epilepsy, septo-optic dysplasia. CC agenesis
	31	ATRX (NM_000489)	XLR	c.109C>T	p.Arg37Ter	Het De novo	P	Mental retardation hypotonic facies syndrome	Childhood	Dysmorphic face
	308	DHDDS (NM_001319959.1)	AD	c.353G>A	p.Arg118Gln	Het De novo	P	Developmental delay and seizures with or without movement abnormalities	Childhood, Tremor	Epilepsy
Dyskinesia	58	FOXG1 (NM_005249.4)	AD	c.506dup	p.Lys170GlnfsTer285	Het De novo	P	Rett syndrome	Infancy, stereotype, chorea, ataxia	Congenital hypotonia, dysmorphic face, microcephaly, exotropia, epilepsy
Tremor	201	FH (NM_000143.3)	AR	c.1108+1G>A c.664T>C	p.Ser222Pro Splicing	Het From father From mother	VUS LP	Fumarase deficiency	Infancy, nystagmus, dystonia, myoclonus	Congenital hypotonia, dysmorphic face, epilepsy, extensive MCD<

a Patients who did not perform the parent tests.

P, pathogenic variant; LP, Likely pathogenic variant; VUS, Variant of uncertain significance.

The identified variants according to the prominent movement disorders are described in Table 2. The variant in most of the patients (*n* = 40/42, 95.2%) was confirmed by parental studies. Two patients in whom parental tests were not done are diagnosed by previously reported pathogenic variants according to the American College of Medical Genetics guideline and phenotype (Li et al., 2017). Thirty-one patients had *de novo* variants. Two patients had inherited the genes from their affected parents with autosomal dominant disorders. Biparental inheritance was confirmed in seven patients with autosomal recessive disorders and asymptomatic maternal carrier status in two patients with X-linked disorders.

Variants in the *GNB1, CACNA1A, TCF4, DHDDS,* and *CTNNB1* genes were detected twice, and the other genes were identified only once each.

### 3.3 Genetic Diagnosis According to Their Movement Phenotypes

#### 3.3.1 Ataxia

In 17 patients with persistent ataxia, the mutation in *GNB1(2)*, *KMT2A, UBE3A, GABRG2, SURF1, TCF4, ITPR1, FRRS1L, SLC2A1, MAST1, MED13L, DYNC1H1, DEPDC5, RTEL1, ATM,* or *EBF3* genes were found to be causative. Congenital hypotonia was the initial presenting sign in most cases, excluding one patient with the *SURF1* mutation. Non-progressive ataxia developed between age 6 and 12 months. The patient with the *SURF1* mutation showed normal development until age 2 years, when ataxia abruptly developed after a febrile illness.

Episodic ataxia was noted in three patients. Two patients with the *CACNA1A* mutations experienced recurrent cerebral infarction and episodic ataxia after febrile status epileptics, beginning in infancy or early childhood. Exercise aggravated the episodic ataxia in the patient with *KCNA1* mutation since age 3 years. Of note, one patient with an *FTL* mutation developed progressive ataxia from age 13 years and was ultimately diagnosed with neurodegeneration with brain iron accumulation 3 (OMIM 606159).

#### 3.3.2 Dystonia

In eight patients with generalized dystonia from infancy, the mutation of *PIGA, TCF4, CHRNG, SLC16A2, KCNQ2, NACC1, CTNNB1*, or *ARSA* gene were found to be causative. The *TOR1A*, *KMT2B,* or *WASHC5* mutations were identified as causative gene in three patients with childhood-onset focal dystonia.

#### 3.3.3 Stereotypy

In five patients with stereotypic movement as the only prominent phenotype, *DHDDS, ARCN1, ZEB2, CTNNB1,* or *MECP2* mutations were identified, while other movement phenotypes accompanied three patients. Patients with SRMMD (short stature, rhizomelic, with microcephaly, micrognathia, and developmental delay; OMIM 617164), or Mowat–Wilson syndrome (OMIM 235730) initially developed congenital hypotonia, followed by stereotypy, observed since ages 6 and 2 years, respectively.

#### 3.3.4 Myoclonus

In three patients with myoclonus as primary phenotype, mutation of *TUBB2B* (OMIM 610031)*, ATRX,* or *DHDDS* (developmental delay and seizures with or without movement abnormalities; OMIM 617836) gene were found to be causative.

#### 3.3.5 Others

One patient with unspecified dyskinesia had *FOXG1* mutation, and a patient with tremor had *FH* mutation. All patients had a history of early-onset congenital hypotonia.

### 3.4 Separated or Combined Movement Phenomenology in Patients With Neurogenetic Disease

**FIGURE 3:**
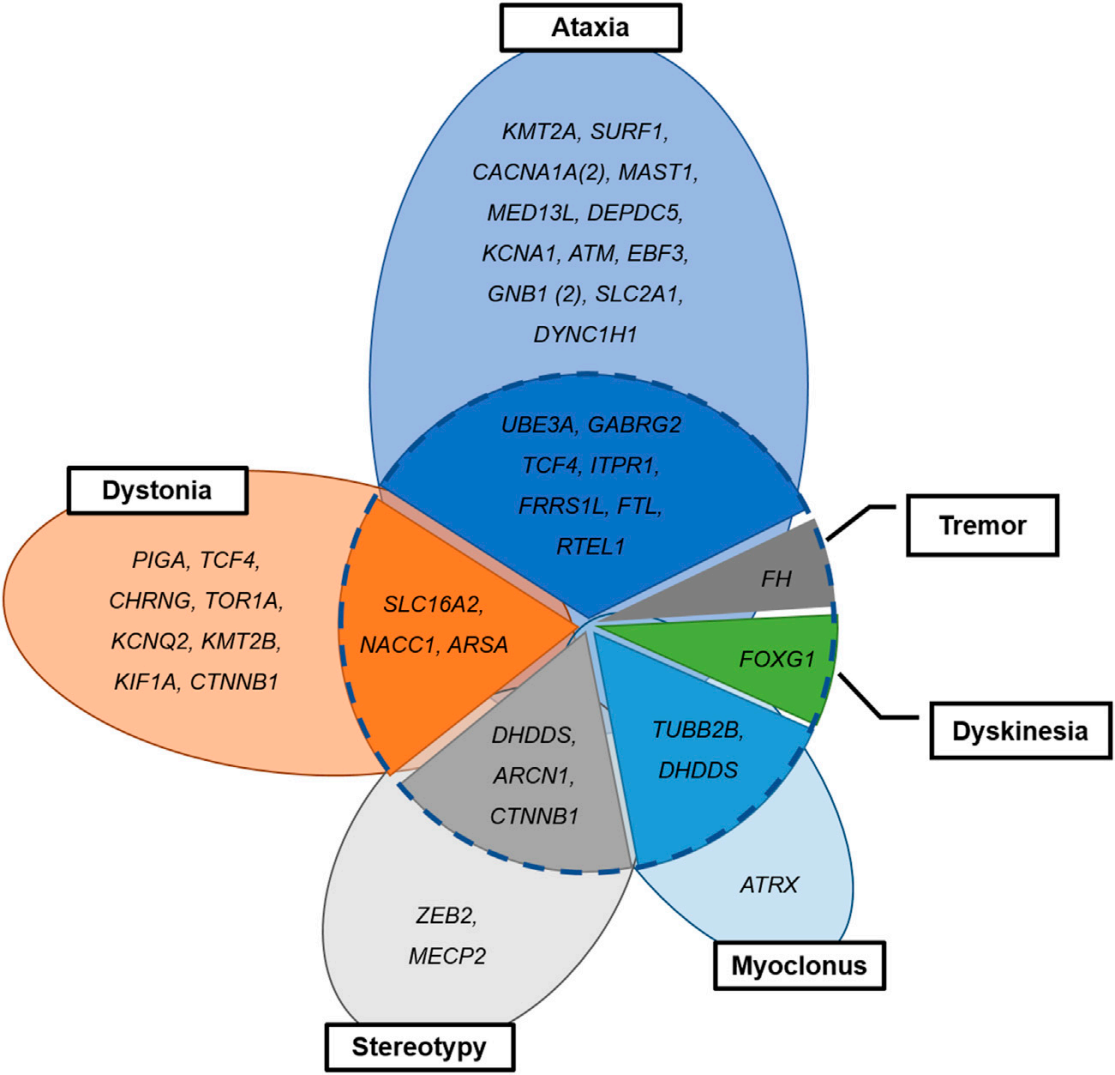
Combined or separated movement phenomenology in patients with neurogenetic disease. Dotted circle: combined (inside) and separate phenotypes (outside).

Seventeen patients (*n* = 17/42, 40.5%) with neurogenetic disease had complex movement phenomenology (Figure 3). Combined movement phenotypes were noted in seven patients (*n* = 7/19; 36.8%) with ataxia and nystagmus, three patients (*n* = 3/11; 27.2%) with dystonia, three patients (*n* = 3/5; 40.0%) with stereotypy, and two patients (*n* = 2/3; 66.6%) with myoclonus. Two patients with unspecified dyskinesia or tremor had variants in the *FOXG1* or *FH* genes, combined with other movement phenotypes. Interestingly, among two patients with *TCF4* mutation, one showed dystonia only and other represented ataxia and myoclonus. The two patients with *DHDDS* mutation showed stereotypy with dystonia and myoclonus with tremor, respectively.

### 3.5 Genetic Relationship Between Movement Disorders and Other Systemic Manifestations

**FIGURE 4:**
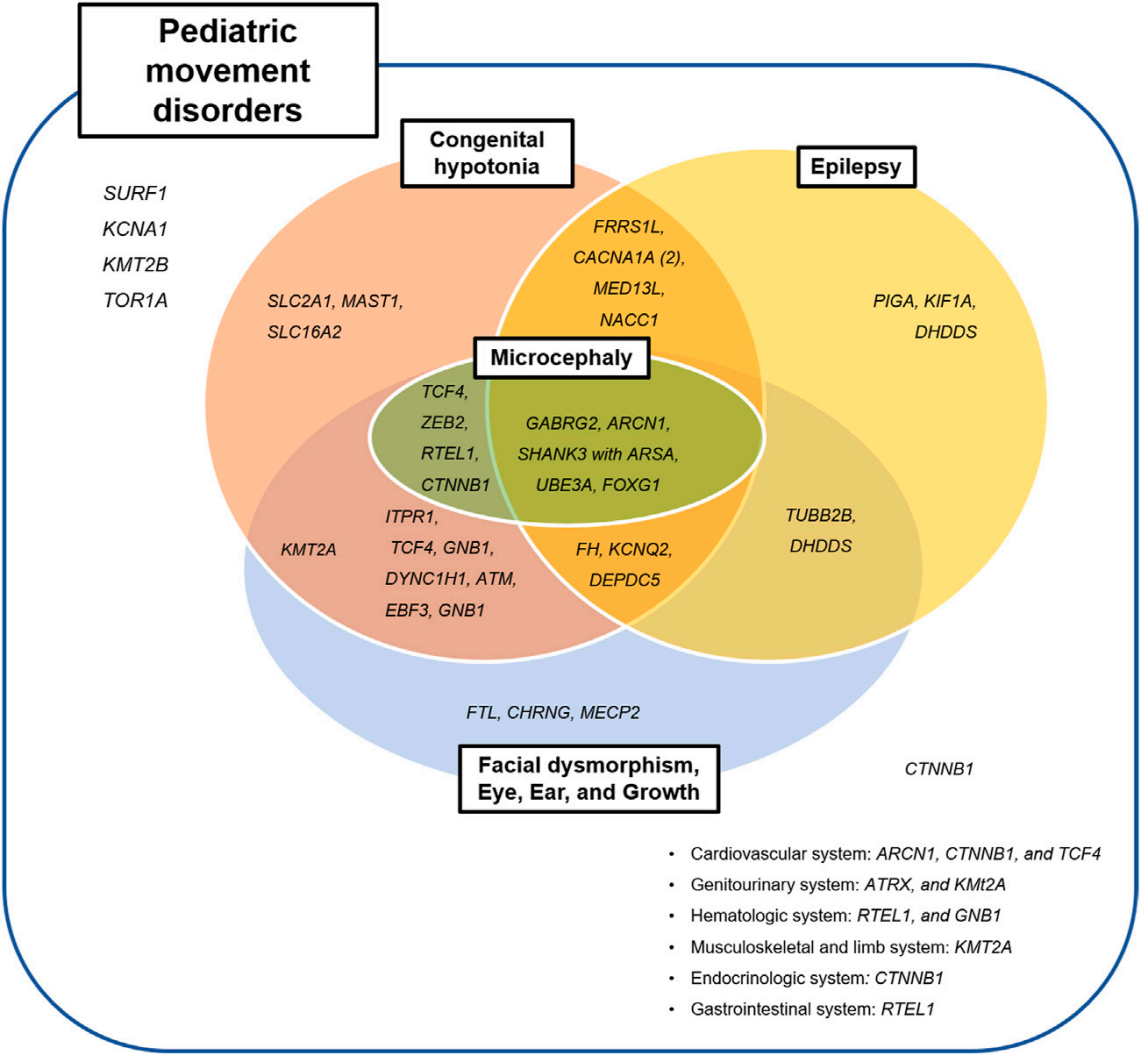
Clinical categories of identified pathogenic variants.

Thirty-eight patients who diagnosed with neurogenetic disease had other neurologic or systemic manifestation, except three patients with variants in *KCNA1, KMT2B, and TOR1A* genes (Figure 4); The patient with the *KCNA1* mutation had episodic ataxia alone, and those with the *KMT2B* or *TOR1A* mutations showed dystonia only.

The most commonly associated systemic manifestation was dysmorphic face, presented in 18 patients (*n* = 18/42, 42.9%). Three patients with the *CHRNG, MECP2,* or *ATRX* mutations had dysmorphic face only, without other neurologic manifestations. Commonly associated neurologic symptoms included congenital hypotonia (*n* = 28/42; 66.7%) and epilepsy (*n* = 18/42; 42.9%). Congenital hypotonia and epilepsy were not observed in two patients with *SURF1* or *FTL* mutations, who had presented with ataxia and childhood-onset cerebellar abnormalities.

#### 3.5.1 History of Congenital Hypotonia

Congenital hypotonia was accompanied in two-third of patients (*n* = 28/42; 66.7%); 16/19 patients (84.2%) with ataxia (*GNB1, KMT2A, UBE3A, GABRG2, CACNA1A (2), TCF4, ITPR1, FRRS1L, SLC2A1, MAST1, MED13L, DEPDC5, RTEL1, ATM, or EBF3*), 6/11 patients (54.5%) with dystonia (*TCF4, SLC16A2, KCNQ2, NACC1, CTNNB1*, or *ARSA*), 2/5 patients (40.0%) with stereotypy (*ARCN1* or *ZEB2*), 2/2 patients (100%) with spasticity (*GNB1* or *DYNC1H1*), 1/1 patient (100%) with unspecified dyskinesia (*FOXG1*), and 1/1 patient (100%) with tremor (*FH*).

#### 3.5.2 Concomitant Epilepsy

Epilepsy was present in almost half of patients (*n* = 18/42; 42.9%); 7/17 patients (41.2%) with ataxia (*CACNA1A (2), UBE3A, GABRG2, FRRS1L, MED13L,* or *DEPDC5*), 5/11 patients (45.5%) with dystonia (*PIGA, KCNQ2, NACC1,WASHC5*, or *ARSA*), 2/5 patients (40.0%) with stereotypy (*DHDDS* or *ARCN1*), 2/3 patients (50.0%) with myoclonus (*TUBB2B* or *DHDDS*), 1/1 patient (100.0%) with unspecified dyskinesia (*FOXG1*), and 1/1 patient (100.0%) with tremor (*FH*). The 10/18 patients with epilepsy and genetic diagnosis (55.5%) were diagnosed as having epileptic encephalopathy spectrum including early infantile epileptic encephalopathy, epileptic spasms, and Lennox-Gastaut syndrome. Other eight patients without epileptic encephalopathy also received more than two anti-seizure medications.

Genes previously been reported to cause epilepsy with movement disorders (*KCNA1, KMT2B, CACNA1A, FRRS1L, KCNQ2, SLC2A1, UBE3A, GABRG2, GNB1, FOXG1,* or *MECP2*) were also observed in our study; however, epilepsy was not associated with *KCNA1, KMT2B, SLC2A1, GNB1,* or *MECP2* in our cohort. On the other hand, ten additional genes were associated with epilepsy in our study (*MED13L, DEPDC5, PIGA, NACC1, ARSA, KIF1A, DHDDS, TUBB2B, ARCN1,* or *FH*), for which there is no previously reported association with epilepsy-dyskinesia disorders (Figure 4).

#### 3.5.3 Clinical Management According to Genetic Diagnosis

Genetic diagnosis changed the clinical care of five patients (*n* = 5/75, 6.7%). They were treated with targeting the identified abnormalities at the molecular level with a ketogenic diet (with a variant in *SLC2A1*), acetazolamide (with two variants in *CACNA1A*), and vitamin E and alpha-lipoic acid (with a variant in *ATM*). Cancer screening was performed in two patient with the *FH* and *ATM* mutation (Chan et al., 2017; Garg and Rabban, 2021). The patient with a variant in SLC2A1 showed significant improvement of their ataxia after ketogenic diet recommendation and was able to walk alone at 5 years of age. The two patients with variants in *CACNA1A* showed decreased episodic ataxia events after acetazolamide treatment. The patient with the *ATM* variant reports some improved effect on their ataxia after alpha-lipoic acid administration. There were no abnormal cancer related findings after screening in the two patients with the *FH* and *ATM* variants. Annual follow-up was scheduled.

## 4 Discussion

Movement disorders sometimes can be key features in the differential diagnosis of neurogenetic disorders; however, their phenotypes have received less attention owing to their vague and complex features.

In the current study, we described the clinical and genetic features of the movement phenotypes presenting in a cohort of pediatric patients with neurogenetic disorders. Despite the small size of patient cohort, WES revealed high diagnostic rate. Patients with a genetic diagnosis more often accompanied the movement phenotype of dystonia, congenital hypotonia, and epilepsy. Importantly, our study dissected the complexity between diverse phenotypic and genetic heterogeneity, offering some new clinical and genetic insights. Additionally, we provide the chance to patients-tailored treatment according to the genetic diagnosis in patients with movement phenotype.

We identified 42 variants in 37 different genes, which yielded a diagnostic rate of 56.0%. Six studies have identified genetic disorders using NGS, including targeted or WES in adults (Neveling et al., 2013; van Egmond et al., 2017; Montaut et al., 2018; Reale et al., 2018) and pediatric patients (Cordeiro et al., 2018; Graziola et al., 2019) with movement disorders, and the diagnostic yield varied from 11.3 to 51%. The rate of diagnosis was higher in studies with a pediatric cohort (28–51%) than with adult patients (11–22%). In addition, studies using WES ((Neveling et al., 2013; Cordeiro et al., 2018), 20–51%) yielded a higher diagnostic rate than those using panel gene testing ((Montaut et al., 2018; Reale et al., 2018; Graziola et al., 2019), 11–28%).

Pediatric movement disorders show complex, evolving phenotype patterns, and are frequently observed as part of multiple neurological or systemic manifestations. For these reasons, the genetic diagnosis of pediatric movement disorders is challenging. In our study, multidisciplinary approach to dissect the patient phenotypes, systemic as well as neurological, by a pediatric neurologist and clinical geneticist, and the application of specific NGS technology using an artificial-intelligence-based, automated variant-priority analysis system (Seo et al., 2020) might have helped to achieve a higher diagnostic rate. By broadening the association-linkage between human phenotypes and genes, this automated system might have been able to detect variants that could otherwise have been missed.

Previously studies have noted differences in the distributions of the causing gene between adults and pediatric cohorts. The *PARKIN, LRRK2, GBA, PLA2G6, VSP13A, and ATP13A2* genes have been considered the major causative genes in adult populations (van Egmond et al., 2017; Montaut et al., 2018; Reale et al., 2018), whereas the causative genes were diverse in pediatric cohorts; The 14 dyskinesia patients with the *PRRT2* or *SLC2A1* mutations (Graziola et al., 2019), 6 dystonia patients with the *ATP1A3* mutations (Graziola et al., 2019), and 4 ataxia with the *STXBP1* mutations (Cordeiro et al., 2018). In our pediatric patients, the genetic spectrum was more diverse and complex, and the main movement phenomenology was heterogeneous even among the same gene-defect carriers (*GNB1, TCF4, DHDDS,* or *CTNNB1* genes).

In our patient cohort, the most common phenomenologies were ataxia and dystonia. Patients with dystonia had a higher genetic risk profile compared with other movement phenotypes (Graziola et al., 2019). In contrast with previous studies (Cordeiro et al., 2018; Graziola et al., 2019), only a small proportion of patients had dyskinesia (5.3%) and no patient was identified carrying the *PRRT2* mutation which is a well-known, most-common cause of the paroxysmal dyskinesia (Erro et al., 2014; De Gusmao and Silveira-Moriyama, 2019). We performed a single gene test for *PRRT2* for patients with paroxysmal dyskinesia who were not eligible for WES, which may be a reason for these results.

In line with other studies (Cordeiro et al., 2018; Montaut et al., 2018; Graziola et al., 2019), half of our patients with a neurogenetic diagnosis had combined movement phenotypes. Traditionally, genetic dystonia has been divided into three main categories according to the combined movement phenotype: isolated dystonia, dystonia combined with myoclonus or parkinsonism, and complex, in which dystonia is a feature of complex neurological syndromes (Pearson and Pons, 2019). In our study, importantly, these combined phenotypes were noted in patients affected by the neurodegenerative diseases caused by the *SLC16A2, ARSA*, *FOXG1, or FH* mutations (Figure 3), which warrants the monitoring of disease progression in the affected patients.

Two third of our patients coexisted with other neurologic manifestation, and it is of note that congenital hypotonia were highly detected in the genetically diagnosed patients. This could be explained by the fact that the brain underdevelopment contributes to hypotonia development during neonatal and infantile periods and then, the motor symptoms such as movement phenomenologies evolve with age as the brain develops, even when the underlying pathology is non-progressive (Pearson and Pons, 2019).

Additionally, epilepsy was also a commonly associated neurologic manifestation in diagnosed patients. Recently, the researchers are becoming aware of the increasing number of neurogenetic comorbid conditions presenting with both epilepsy and atypical movements (Papandreou et al., 2020). These disorders within the ‘genetic epilepsy-dyskinesia’ spectrums are clinically and genetically heterogeneous from isolated movement disorders to severe infantile epileptic encephalopathy (de Gusmao and Waugh, 2018; De Gusmao and Silveira-Moriyama, 2019; Papandreou et al., 2020). Over 60 genes have been reported as associated with epilepsy-dyskinesia phenotype (de Gusmao and Waugh, 2018; De Gusmao and Silveira-Moriyama, 2019; Papandreou et al., 2020), and the list is expanding. In our study, *MED13L, DEPDC5, PIGA, NACC1, ARSA, WASHC5, DHDDS, TUBB2B,* or *ARCN1* can be added to this category (Figure 4).

Importantly, the genetic diagnosis changed the clinical care for five patients (6.7%) in our study. Four patients received targeted molecular therapy. For the patient with a mutation in the *ATM* gene mutation, encoding ATM serine/threonine kinase protein which is involved in the DNA repair machinery, antioxidants including vitamin E and alpha-lipoic acid were given to reduce the increased oxidative stress (Maciejczyk et al., 2017; Blignaut et al., 2021). For the patient with *SLC2A1* mutation, the ketogenic diet, the first line treatment for GLUT1 deficiency syndrome, were offered to produce ketone bodies as an alternative energy source for brain metabolism and to bypass the *GLUT* defect (Koch and Weber, 2019). The *CACNA1A* mutation results in reduced calcium current and decreased inhibitory effects of Purkinje cells. Acetazolamide responsiveness is a hallmark of the disease and about 50–75% of patients show rapid improvement in episode frequency and severity (Kotagal, 2012). We performed tumor surveillance for the patient with a *FH* (HLRCC; OMIM #150800) and *ATM* mutations.

The clinical features and course of movement phenotypes in pediatric patients with neurogenetic disorders are diverse, difficulting the clinical classification of pediatric movement disorders. Due to the diverse and complex range of phenotypic and genetic heterogeneities, genome sequencing (WES or WGS) should be considered for patients with movement disorders. Additionally, WES/WGS may be preferred over panel-gene testing for revealing and understanding this complexity. Since most patients in our cohort underwent other genetic testing including karyotyping, CMA, or single gene sequencing prior to WES, the diagnostic yield of WES can be misleading and the exact superiority of WES could not be assessed. However, as shown in our study, the early application of WES with its rapid interpretation by automated system yields the acknowledgeable diagnostic rate and physicians can overcome the limitations of the usual phenotype-driven strategies and avoid the long journey for diagnosis. Furthermore, phenotypic reanalysis after genetic confirmation help to understand the expanding clinical spectrum of a genetic disease and identification of the patients in their early disease state, despite not exhibiting the full spectrum of the disease.

## Data Availability

The original contributions presented in the study are publicly available. This data can be found here: https://www.ebi.ac.uk/ena/browser/view/PRJEB51291.
